# Mentalized affectivity: A new model and assessment of emotion regulation

**DOI:** 10.1371/journal.pone.0185264

**Published:** 2017-10-18

**Authors:** David M. Greenberg, Jonela Kolasi, Camilla P. Hegsted, Yoni Berkowitz, Elliot L. Jurist

**Affiliations:** 1 Department of Clinical Psychology, The Graduate Center and City College of New York, City University of New York, New York, United States of America; 2 Department of Psychiatry, University of Cambridge, Cambridge, United Kingdom; 3 Department of Psychology, City College of New York, City University of New York, New York, United States of America; Institut d'Investigacio Biomedica de Bellvitge, SPAIN

## Abstract

Here we introduce a new assessment of emotion regulation called the Mentalized Affectivity Scale (MAS). A large online adult sample (*N* = 2,840) completed the 60-item MAS along with a battery of psychological measures. Results revealed a robust three-component structure underlying mentalized affectivity, which we labeled: **Identifying** emotions (the ability to identify emotions and to reflect on the factors that influence them); **Processing** emotions (the ability to modulate and distinguish complex emotions); and **Expressing** emotions (the tendency to express emotions outwardly or inwardly). Hierarchical modeling suggested that Processing emotions delineates from Identifying them, and Expressing emotions delineates from Processing them. We then showed how these components are associated with personality traits, well-being, trauma, and 18 different psychological disorders (including mood, neurological, and personality disorders). Notably, those with anxiety, mood, and personality disorders showed a profile of high Identifying and low Processing compared to controls. Further, results showed how mentalized affectivity scores varied across psychological treatment modalities and years spent in therapy. Taken together, the model of mentalized affectivity advances prior theory and research on emotion regulation and the MAS is a useful and reliable instrument that can be used in both clinical and non-clinical settings in psychology, psychiatry, and neuroscience.

## Introduction

In early life, emotions seem to have a life of their own, but as cognition develops, people devise strategies for managing their emotions that they strive to use throughout adulthood [1]. Emotion regulation is one of the fasting growing areas in psychology [2] and several theories have been developed to help our understanding of the regulatory processes that are adopted when dealing with emotions [3–6]. However, there are currently a limited number of emotion regulation measures in use, and they often target only specific aspects of emotion regulation, such as deficits or dysfunction. Further, current models of emotion regulation do not take into account mentalizing (‘theory of mind’) and the ability to draw from prior and present contexts to understand and manage emotions—both of which we argue are central to understanding and regulating emotions [7]. The present research introduces a new model and assessment of emotion regulation based on the theory of *mentalized affectivity* (MA), which integrates prior theory and research on emotion regulation and mentalization [8]. By using this new assessment in a large sample, we show how three aspects of mentalized affectivity (Identifying, Processing, and Expressing) are linked to personality, well-being, trauma, clinical diagnoses (including neurobiological, mood, and personality disorders) and psychological treatment histories. As will be shown, the virtue of this new perspective on emotion regulation is its breadth, with its measure covering a wider terrain than other measures in this area.

### Theoretical background

Emotion regulation is a wide-ranging term that describes explicit and implicit processes that involve monitoring, evaluating, altering, and modulating emotions [9–12]. Mentalization is the cognitive and affective ability to understand the thoughts and feelings of ourselves and others [13–15]. Over the past decades, theory, research, and measures have been developed in each of these areas, in both clinical and non-clinical settings. Except for a few exceptions, these constructs have been investigated and discussed independently in the psychological literature [16]. This is surprising considering both are fundamental to how people understand, experience, and respond to their emotions. Indeed, the process of emotion regulation involves being aware, understanding, and identifying one’s thoughts and feelings (i.e. mentalization), prior to, during, and after the refining and modulating of the emotion.

Jurist [7–8] proposed a novel perspective on emotion regulation called the theory of mentalized affectivity (MA), which takes mentalization into account in the regulatory process. The theory argues that affectively regulating (managing, altering, or changing) an emotion relies on the capacity for mentalization. It argues that emotions are not just adjusted in a regulatory process, but that they are also revalued in meaning. This more sophisticated aspect of emotion regulation requires the ability to reflect on one’s thoughts and feelings and to mentalize about the factors that may influence the emotion, such as childhood experiences or the present situation or context a person is in. This in turn helps to inform a person’s understanding about their emotions and how to anticipate future situations.

Within MA theory, Jurist [8] proposed three delineated aspects that are part of a concentric process of emotion regulation. The first is *Identifying* emotions, which in its most basic form involves labeling emotions. It also includes deeper complexities that involve making sense of emotions in the context of one’s personal history and exploring the meaning of emotions (e.g. Why am I feeling this way?). The second aspect that follows Identifying emotions, is *Processing* them. Processing involves modulation/regulating emotions. This includes changing the emotion in some way, such as by duration or intensity. The third process that follows Processing is *Expressing* emotions. Expressing involves the spectrum of communicating one’s thoughts and emotions from inwardly to outwardly. A person’s prior history influences every aspect of emotional experience from identifying and processing to expressing. Further, these elements are tied to a person’s sense of agency with emotions—with identifying, there is the dawning of a sense of agency, with modulating the actualization of agency, and with expressing, the results or manifestations of agency.

**Fig 1:**
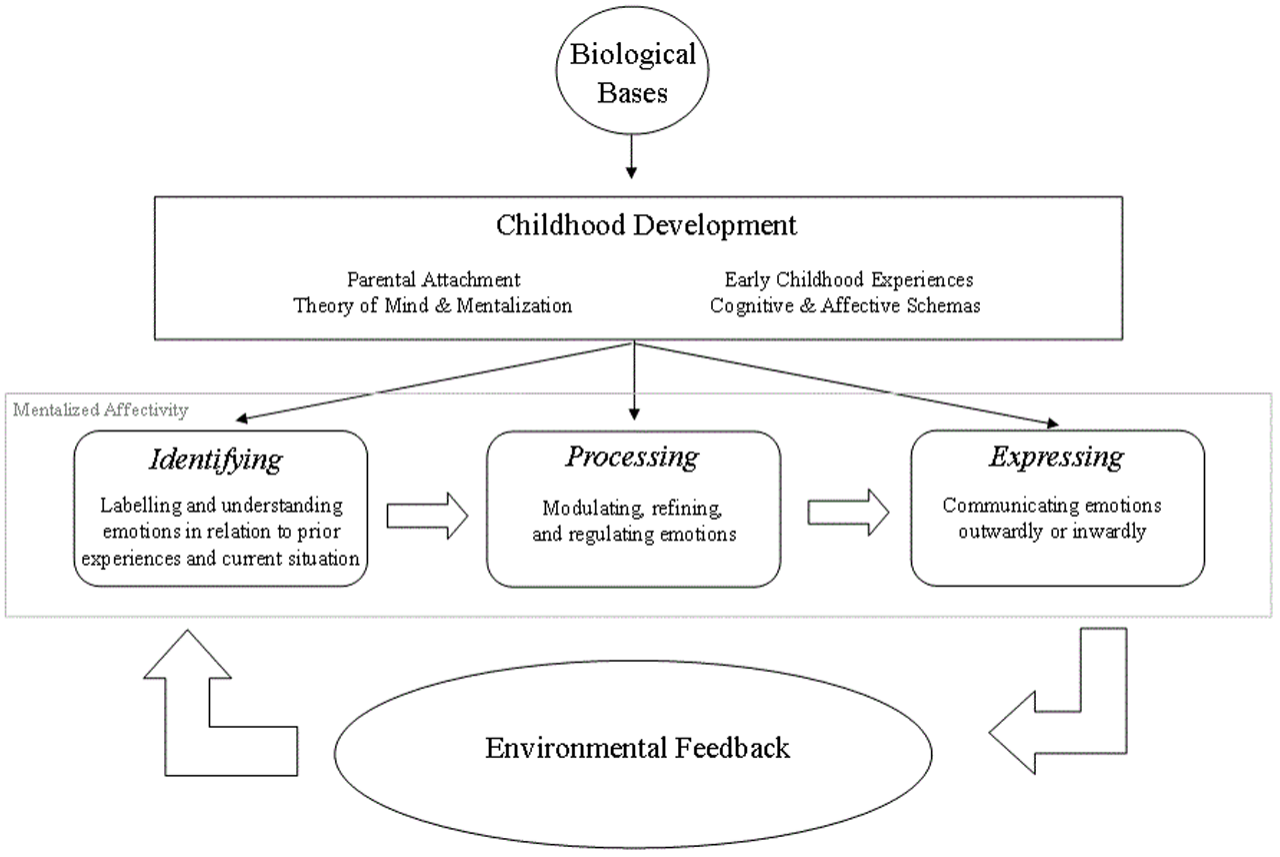
Flow chart of MA theory.

A flow chart of MA theory is visually displayed in Fig 1. The chart begins with biological bases (e.g. genetics and dispositional traits) at the top. Following biological bases is childhood development. Specifically, within childhood development is attachment formation, the development of ‘theory of mind’ and mentalization [17], and the development of cognitive and affective schemas [18]. After biological bases and childhood development are the three delineated aspects of mentalized affectivity: Identifying, Processing, and Expressing. Expressing emotions leads to interpersonal interactions with others and the environment—including feedback from others and the experience of new situations and events (labeled in the diagram as “environmental feedback”). This feedback and interplay with the environment is integrated with prior schemas that impact mentalized affectivity. Further elaboration of MA theory can be found in Jurist (2005; in press). In the present research, we aimed to develop an assessment that would capture these three elements of emotion regulation.

### Previous measures of emotion regulation and mentalization

There are several widely used scales used on emotion regulation, and they converge and diverge with MA theory in various ways. The Difficulties in Emotion Regulation Scale [4] is a 36-item self-report measure that captures six components of dysregulation in emotion regulation: Non-acceptance of emotional responses (“Nonacceptence”); Difficulties engaging in goal directed behavior (“Goals”); Impulse control difficulties (“Impulse”); Lack of emotional awareness (“Aware); Limited access to emotion regulation strategies (“Strategies”); and Lack of emotional clarity (“Clarity”). One limitation of the scale is that it doesn’t focus on the entire spectrum and multi-dimensional space of emotion regulation—rather it focuses primarily on deficits or difficulties. Further, it predominantly focuses on emotion regulation that occurs during or after an individual feels a negative affect. Specifically, 27 of the 36 items begin with the phrase “When I’m upset,…”. “Aware” and “Clarity” most closely resembles the negative polar ends of Identifying from MA theory, and “Strategies” most closely resembles the negative polar ends of Processing. “Nonacceptence”, “Goals”, and “Impulse” lie outside of the scope of MA theory because they largely describe behavioral responses to emotions, rather than the process of regulating, modulating, or refining them.

Another widely used measure of emotion regulation is the Emotion Regulation Questionnaire (ERQ) aimed to capture antecedent- and response-focused emotion regulation strategies, which are based on the process model of emotion regulation [19]. A virtue of the ERQ is that in contrast to the DERS, it looks at both adaptive and maladaptive styles of emotion regulation. The 10-item measure captures the factors: Reappraisal and Suppression (representing by antecedent-focused and response-focused strategies respectively). The Reappraisal factor includes items such as “I control my emotions by changing the way I think about the situation I’m in” and “When I want to feel less negative emotion, I change the way I’m thinking about the situation”. The Suppression factor includes items such as “I control my emotions by not expressing them”, “When I am feeling negative emotions, I make sure not to express them”, and “I keep my emotions to myself”. The ERQ correlates with big five personality traits, and seeks to measure the regulation of emotions, emphasizing control and focusing, in particular, on change. In relation to MA theory, the Reappraisal factor captures Processing and the Suppression factor captures the negative polar end of Expressing. However, there are no items from the ERQ that represent elements of Identifying emotions.

Hofmann and Kashdan’s [20] Affective Style Questionnaire (ASQ) is a 20 item self-report measure that builds on the notion that emotions can be regulated according to different styles. The authors identify three styles, extending the ERQ’s distinction between cognitive reappraisal and expressive suppression, by distinguishing among concealing (suppression), adjusting (cognitive reappraisal) and tolerating. The ASQ captures three factors: Concealing, Adjusting, and Tolerating. Findings from the ASQ lend support to the findings from the ERQ that concealing is a maladaptive strategy for coping with negative affect. The ASQ adds an emphasis on how emotions are tolerated, particularly emotions of distress, and suggests that personality has an impact on how someone regulates their emotions. In relation to MA theory, the Adjusting factor captures Processing and the Concealing factor captures the negative polar end of Expressing. As with the ERQ, there are no items from the ASQ that represent elements of Identifying emotions.

A more recent measure is the Flexible Regulation of Emotional Expression Scale (FREE) by Burton and Bonnano [21]. The measure questions emotion regulation scales that contrast the desirability of cognitive reappraisal to the liability of expressive suppression, construing the latter in terms of frequency of behavior, rather than ability. While conceding that the FREE does not represent one dimension of expressive regulation, the authors draw attention to a previously unidentified merit of the capacity to suppress expression. Notably, the authors do not speculate where ability for expression comes from; nor do they focus attention on verbal expression per se. At the heart of the measure is an appreciation for both expression and constraint, and for the notion that flexibility of expression is a buffer against stress. The measure captures two second-order factors (Enhance and Suppress) and four first-order factors (Enhance Positive, Enhance Negative, Suppress Positive, and Suppress Negative). All scenarios of the questionnaire involve aspects of expressing or concealing an emotion being felt, therefore, each of the FREE factors link to the Expressing dimension of MA theory.

There are several measures on emotion regulation that are less widely used [3]. The Emotional Regulation of Others and Self (EROS) [22] captures four factors related to improving and worsening affects in both the self and others. The Cognitive Emotion Regulation Questionnaire (CERG) [23] measures how people think and feel in response to prior experiences. It captures nine factors: Self-blame; Acceptance; Rumination; Positive refocusing; Refocus on planning; Positive reappraisal; Putting into perspective; Catastrophizing; and Blaming others. A limitation of this scale is that it only captures cognitive aspects of regulation. Further, the measure only captures how someone responds to a negative life events and how they reflect on those experiences—not necessarily emotions (“I think about the mistakes I have made in this matter” and “I think that the situation also has its positive sides”) [24]. The Rumination factor does, however, associate with aspects of Identifying in MA theory. There are also tangential scales to emotion regulation that are worth noting such as the Cope Inventory (COPE) [25] and the Coping Styles Questionnaire (CSQ) [26] that do not capture emotion regulation, but rather individual differences in how people cope (e.g. avoidance or problem-focused) with different situations rather than focusing on emotions.

Measures on mentalization are also relevant for emotion regulation. The newly developed self-report Reflective Functioning Questionnaire (RFQ) [27] captures mentalization (previously measured with interview-based questionnaire [28], which consists of both interpersonal and self-reflective components that help to interpret the thoughts and feelings of another and oneself. The 70-item RFQ captures two factors: Certainty and Uncertainty. These factors most closely resemble the Identifying aspects of MA theory, but do not capture aspects of Processing or Expressing. In addition, the RFQ assumes, but does not actually measure how previous life history and experience may inform how we identify and understand emotions. Finally, the RFQ focuses on mentalization in general, rather than on emotions.

Other measures that are similar to mentalization and the Identifying aspect of MA theory are scales on alexithymia and to a lesser extent, scales on rumination. For example, the Toronto Alexithymia Scale (TAS) [29] captures three factors related to Identifying emotions: difficulty in describing feelings, difficulty in identifying feelings, and emotionally-oriented thinking. In terms of ruminations, scales such as the Rumination Inventory (RI) [30] captures several factors including Tendency Toward Repetitive Thought; Tendency to Engage in Mental Rehearsal of Future and Past Events; and Distractibility. Though rumination measures capture aspects of a process involved in labeling emotions and thinking about events that influence them, they do not explicitly capture aspects of Identifying, and lie tangential to mentalized affectivity and emotion regulation.

Another related construct is empathy, which involves both the cognitive and affective ability to understanding and respond to the emotions of others [31]. It involves an aspect of cognitive empathy (also referred to as theory of mind or mentalization) where a person puts themselves in another’s shoes to identify their thoughts and emotions. It also involves the affective aspect of responding to others with an appropriate emotion by processing the situation another person is in and how it makes one feel (including feeling sympathy in response to another person’s suffering). Empathy also involves elements of social interaction. The Empathy Quotient [31] because it captures scores on these three elements. Specifically, it measures facets of Cognitive Empathy, Emotional Reactivity (i.e. affective empathy), and Social Skills. Empathy and its three facets resemble the three MA components of Identifying, Processing, and Expressing, however, they are directed outward to people and objects rather than inward to the self.

**Fig 2:**
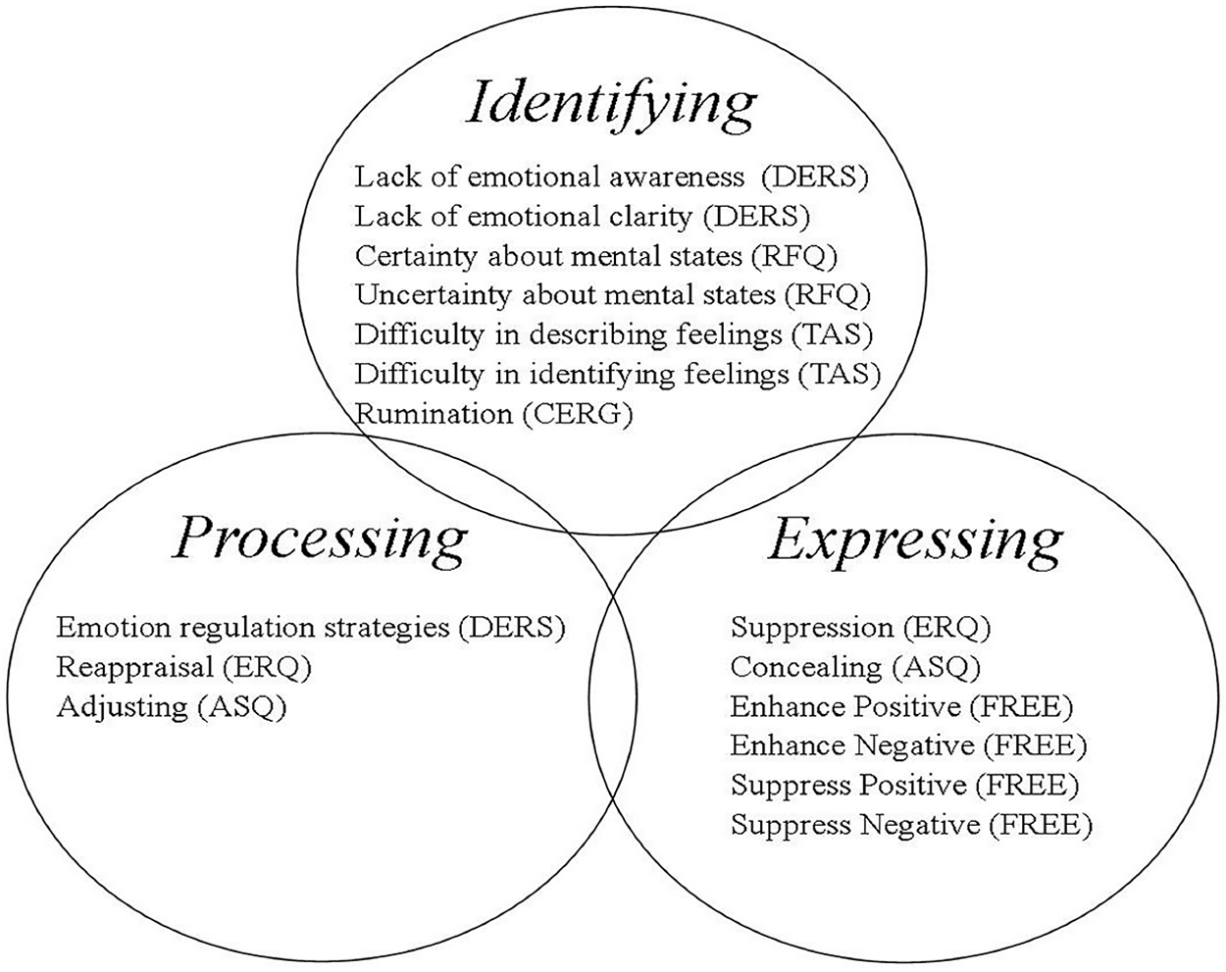
Diagram of converging and diverging scales from prior measures related to emotion regulation.

As can be seen, prior measures on emotion regulation, coping, mentalization, alexithymia, and rumination capture aspects of MA theory, but none capture all three components, and they do not address aspects of understanding prior and present contexts that inform emotion regulation. We have displayed this comparison of measures visually in Fig 2. The diagram highlights two important conclusions: 1) all three aspects of mentalized affectivity have been captured in prior measures on emotion regulation, giving precedent for studying them in relation to emotion regulation and 2) none of the measures reviewed captures all three aspects of mentalized affectivity within in a single instrument which gives us further rationale to develop a single measure that captures all three.

### Aims

The overarching goal of the present research was to establish a new multi-dimensional assessment of emotion regulation based on MA theory and to build a model in which we can understand MA in relation to personality, well-being, psychopathology and psychological treatment. Specifically, we aimed to:

1. develop a novel multi-dimensional self-report assessment of emotion regulation based on MA theory (part 1)
2. examine the latent and hierarchical structure of assessment (part 1).
3. test the assessment’s reliability and validity (part 1);
4. examine the psychological correlates of mentalized affectivity including demographics, personality, trauma, and well-being (part 2);
5. test how mentalized affectivity is differentiated across 18 psychological disorders (e.g. Autism, Borderline Personality Disorder, Bipolar Disorder, Post-Traumatic Stress Disorder, Generalized Affectivity Disorder, Depression) (part 3);
6. examine how mentalized affectivity is linked to prior psychological treatments (e.g. CBT, Mindfulness-Based Therapy, and Psychotherapy) (part 3).

We had several hypotheses prior to analysis. First, we predicted that the newly developed scale would reduce into three distinct factors based on MA theory. Second, we predicted that the MA components would correlate with personality and well-being in unique ways. We hypothesized that Identifying would be positively correlated with Openness to Experience, Processing would be negatively correlated with Neuroticism, and Expression would be positively correlated with Extraversion. We predicted that all three MA components would be positively correlated with life satisfaction. We also predicted that Processing would be lower for most psychological disorders including mood and anxiety disorders, and that all three MA factors would be elevated for those with prior psychological treatment.

## Methods

### Participants and procedure

Data was collected online via a website hosted by Qualtrics where participants can take a variety of psychological tests on music, personality, and social psychology (e.g. the Big Five personality traits) in exchange for feedback about their scores. Providing feedback to participants, was our attempt to motivate participants to complete the questionnaire accurately and carefully. Because the data was collected was online, we ensured that all responses came from different IP addresses and only included those participants who completed all items of the measures taken. A total of 2,840 participants completed a test battery that included the Mentalized Affectivity Scale (MAS) and measures on personality and well-being. Subsamples completed additional measures on trauma, empathy, clinical diagnoses, and prior history of psychological treatment. Of those who indicated, 901 (42%) were male, 1180 (55%) were female, 29 (1%) were transgender, and 24 (1%) indicated "other". Participants ranged in age from 18 to 65 with a mean of 31.58 (*SD* = 11.90). Of those who indicated, 1,530 (72%) were White Caucasian, 36 (2%) were African-American or Black, 82 (4%) were Chinese, 81 (4%) were Latino, and 100 (5%) were mixed. The de-identified data is available in supporting information [S1 Dataset].

### Test development

Items for the MAS were generated by four experts, who had previously received weekly training for a year on the emotion regulation and mentalization. In step 1, items were generated based on six different categories and subcategories aligned with the theory of mentalized affectivity: identifying basic affects, identifying complex affects, processing: modulating, processing: refining, outward expressing, and inward expressing. A seventh category of items was developed to include items related to the ability to reflect how present emotions relate to prior and present contexts and experiences (e.g. during childhood). In step 2, two of the judges refined the initial list of items to 76 by removing redundant and poorly worded items.

### Convergent measures

The EQ is a 60-item self-report questionnaire that measures cognitive and affective components of empathy. Of the 60 items, 20 are filler leaving a total of 40 items that measure empathy directly. To prevent participant fatigue, the 20 filler items were excluded, and therefore participants were only presented with 40 items. Participants were asked to indicate their degree of agreement for each statement on a four point scale (*strongly disagree*, *slightly disagree*, *slightly agree*, or *strongly agree*). For positively poled items, two points are given for strong agreement and one point is given for slight agreement. For negatively poled items, two points are given for strong disagreement and one point is given for slight disagreement. Cronbach’s alpha was equal to .90.

### Measures of psychological correlates

#### Personality

Personality was assessed with the Ten-Item Personality Inventory (TIPI) [32]. The TIPI is a very brief measure of the Big Five personality traits and has strong convergent and discriminant validity with related instruments. Participants rated the extent to which each item was characteristic of themselves using a seven-point scale with endpoints at 1 (*extremely uncharacteristic*) to 7 (*extremely characteristi*c). Cronbach’s alphas were equal to .70, .41, .62, .69, and .40 for Extraversion, Agreeableness, Conscientious, Neuroticism, and Openness, respectively. These are consistent with the Cronbach’s alphas found in the study of the development of the TIPI [32].

#### Well-being

General well-being was assessed with the Satisfaction with Life Scale (SWLS) [33]. The SWLS contains five items that measures a person’s judgment about their feelings of life satisfaction. Participants respond to each of the statements using a 7-point scale with endpoints from 1 (*strongly agree*) to 7 (*strongly disagree*). Cronbach’s alpha was equal to .89.

#### Trauma

Recent trauma histories were assessed with a modified version of the Traumatic Events Scale [34]. We did not include items about childhood trauma to avoid participant fatigue and biases related to memory recall. Specifically, participants were asked four questions;

1. “Within the last 3 years, did you experience any other major upheaval that you think may have shaped your life or personality significantly?” Participants responded with one of either three answer choices: “Yes”, “No”, and “Rather Not Say”. If participants indicated “Yes”, then they were asked to provide details via open text.
2. “How traumatic was this?” Participants responded to this item with a seven point scale ranging from 1 (*not at all traumatic*) to 7 (*extremely traumatic*).
3. “When exactly did the event occur?” Participants responded to this item with one of 36 answer choices ranging from *1 month* to *36 months*.
4. “How much did you confide in others about the experience at the time?” Participants responded to this item with a seven point scale ranging from 1 (*not at all*) to 7 (*a great deal*).

#### Questionnaire on clinical diagnoses

Participants were presented with a list of 17 clinical diagnoses with a box for text entry. They were asked to indicate if they had been diagnosed with any of the clinical diagnoses and if so, to indicate the year they were diagnosed, and if they currently experience symptoms by typing “yes” or “no” in the open text box provided. Specifically, participants were asked: “Please indicate below if you have been diagnosed with any of the following conditions. You can select multiple items. For each condition you have been diagnosed with, in the space provided type the age when you were diagnosed and if you currently have symptoms by typing 'yes' or 'no'.”The list included the following: Alexithymia; Anorexia Nervosa; Attention Deficit/Hyperactivity Disorder (ADHD); Bipolar Disorder; Borderline Personality Disorder; Bulimia Nervosa; Depression; Dyslexia; Epilepsy; General Anxiety Disorder (GAD); Narcissistic Personality Disorder; Obsessive-Compulsive Disorder (OCD); Panic Disorder; Posttraumatic Stress Disorder (PTSD); Schizophrenia; Seasonal Affective Disorder (SAD); and Social Anxiety Disorder; Synesthesia.

Participants were separately asked about autism with three questions. They were asked “Have you been formally diagnosed with an Autism Spectrum Condition (ASC) by a professional?” with “Yes”, “No”, and “Other (please specify)”. Second, they were asked “If you answered yes to the question above, please indicate the Autism Spectrum Condition that you have been diagnosed with” and presented with five answer choices: Autistic Disorder (classical autism); Asperger Syndrome (AS); Pervasive developmental disorder, not otherwise specified (PDD-NOS); High-Functioning Autism (HFA); Other (please specify). Third, participants were asked “If you answered yes to the question above, at what age were you diagnosed?” with 90 answer choices ranging from *1 year* to *90+ years*.

#### Questionnaire on psychological interventions

Participants were asked three questions about their prior history with psychological treatment. First, participants were asked, “Are you currently in therapy?” and presented with three answer choices: “Yes”, “No”, and “Rather not Say”. Second, participants were asked “How long have you been in therapy over the course of your life?” with answer choices ranging from “0 years” to “more than 60 years” with half year intervals (e.g. 0, .5, 1.0, 1.5, years). Third, participants were asked, “What kind of therapy were you in? (you can select more than one answers choice)” with answers choices including: I do not know; I have never been in therapy; Cognitive Behavioral Therapy (CBT); Couples Therapy; Creative Arts Therapy; Dialectical Behavior Therapy; Exposure Therapy; Family Therapy; Group Therapy; Mindfulness-based Therapy; Psychoanalysis; and Psychotherapy.

## Results

### Part 1: The structure, reliability, and validity of the MAS

#### Pilot analysis

Principal-components analysis (PCA) with varimax rotation was performed on 76 mentalized affectivity items. The examination of the scree plot suggested an “elbow” at four components suggesting we retain three components. The fourth component was comparatively small and only accounted for an additional 3.8% of the variance. The three components together accounted for 40% of the variance. Item loadings are reported in S1 Table.

One of the limitations of the 76-item version of the MAS is its long length and the potential for participant fatigue when used in the future by researchers. Therefore, in an attempt to eliminate items we conducted two separate steps to psychometrically remove items. First, we removed items that did not have loadings of .40 or above on any of the components. We identified six items (72, 40, 58, 16, 14, and 7) that met this criterion and removed them from the final analyses. Second, we identified items that described the mentalized affectivity of others, such as, “Knowing about the childhood experiences of others helps me to understand their emotions” and “I try to understand the complexity of other people’s emotions.” We deemed these items problematic because of the possibility of subjects to be able to identify and mentalize about other people’s emotions but not their own (as may exist in the case of some personality disorders). We identified ten items (62, 43, 51, 32, 10, 9, 29, 65, 37, and 11) that met this criterion and removed them from the final analysis. However, we kept items that referred to using other people’s emotions to help identify their own, because these still involved the process of identifying emotions about oneself (e.g. item 64: “Thinking about other people’s emotional experiences helps me to think about my own”). After eliminating items using these two steps, 60-items remained for the final analyses.

#### PCA on 60-item MAS

A second principal-components analysis (PCA) with varimax rotation was performed on the final 60 mentalized affectivity items. The Kaiser-Meyer-Olkin Measure of Sampling Adequacy was .95. The examination of the scree plot suggested an “elbow” at four components suggesting we retain three components (S1 Fig). The fourth component was comparatively small and only accounted for an additional 4% of the variance. The three components together accounted for 43% of the variance. Additional analysis on subgroups separated by males and females indicated that the identical scree plots and components structure emerged, suggesting that there were no sex differences driving the component structure.

Table 1 displays the component loadings for the three mentalized affectivity components. As can be seen, the components are transparent and robust with most of the items on each component showing very small secondary loadings. Table 2 displays the between-component correlations. As can be seen, all between-component correlations are below .24 suggesting that each component is independent from one another.

**Table 1:** Component loadings for 60-item MAS.

	Principal Component		
	I	II	III
37. I often look back at my life history to help inform my current emotional state and situation.	**.78**	.03	-.02
18. I often think about my past experiences to help me understand emotions that I feel in the present.	**.75**	-.07	-.03
46. I try to put effort into identifying my emotions.	**.74**	.05	-.02
42. I am curious about identifying my emotions.	**.73**	.01	-.07
33. I try to understand the complexity of my emotions.	**.73**	.09	-.05
70. It’s important to understand the major life events that have had an impact on my behavior.	**.73**	.04	-.03
67. It is helpful to think about how my emotions stem from family dynamics.	**.70**	.06	-.05
16. Knowing about my childhood experiences helps to put my present emotions within a larger context.	**.68**	.06	-.01
1. I often think about how the emotions that I feel stem from earlier life experiences (e.g. family dynamics during childhood).	**.68**	-.18	-.01
47. I can pinpoint childhood experiences that influence the way that I often think and feel.	**.67**	.08	.00
34. It is important for me to acknowledge my own true feelings.	**.67**	.14	-.20
66. I can see how prior relationships influence the relationships that I have now.	**.65**	.14	-.06
8. I am interested in learning about why I feel certain emotions more frequently than others.	**.64**	-.14	-.09
28. It helps me to know the reasons behind why I feel the way that I do.	**.61**	.07	.00
12. Understanding my emotional experience is an ongoing process.	**.61**	-.14	-.02
5. I can see how prior relationships influence my current emotions.	**.61**	.07	-.04
64. Thinking about other people’s emotional experiences helps me to think about my own.	**.59**	.10	-.16
35. I often figure out where my emotions stem from.	**.58**	.41	-.12
29. I am aware of recurrent patterns to my emotions.	**.56**	.21	-.04
69. I rarely think about the reasons behind why I am feeling a certain way.	**-.56**	.00	.09
8. I put effort into managing my emotions.	**.52**	.02	.12
4. I use tools I have learned to help when I am in difficult emotional situations.	**.48**	.23	-.02
68. I am open to other people’s view of me because it helps me to better understand myself.	**.41**	.14	-.06
38. I am open to what others say about me to help me know what I am feeling.	**.40**	.12	-.03
15. It is hard for me to manage my emotions.	.18	**-.71**	-.10
10. When I am filled with a negative emotion, I know how to handle it.	.00	**.68**	.12
23. I am good at controlling my emotions.	-.11	**.67**	.35
13. I am often confused about the emotions that I feel.	.12	**-.66**	.18
41. I am good at distinguishing between different emotions that I feel.	.30	**.64**	-.09
74. I can quickly identify my emotions without having to think too much about it.	.12	**.61**	-.25
11. I often know the reasons why I feel the emotions I do.	.22	**.58**	-.13
75. I am able to understand my emotions within the context of my surroundings.	.31	**.57**	-.13
26. I am good at controlling emotions that I do not want to feel.	-.12	**.57**	.22
14. I am able to adjust my emotions to be more precise.	.13	**.57**	.08
76. I can tell if I am feeling a combination of emotions at the same time.	.37	**.56**	-.14
6. I can still think rationally even if my emotions are complex.	.05	**.56**	.25
25. When I am filled with a positive emotion, I know how to keep the feeling going.	.07	**.54**	-.14
24. When I express my emotions to others, it is usually jumbled.	.00	**-.53**	.38
7. I am able to wait to act on my emotions.	.06	**.52**	.45
43. If a feeling makes me feel uncomfortable, I can easily get rid of it.	-.18	**.51**	.16
39. People get confused when I try to express my emotions.	.03	**-.50**	.35
32. It takes me a while to know how I am really feeling.	.09	**-.49**	.35
22. I am good at increasing emotions that I want to feel more.	.13	**.48**	-.15
17. It is easy for me to notice when I am feeling different emotions at the same time.	.39	**.47**	-.08
21. I can easily label “basic emotions” (fear, anger, sadness joy and surprise) that I feel.	.28	**.46**	-.09
3. I am good at understanding other people’s complex emotions.	.34	**.42**	-.22
71. I am not aware of the emotions I’m feeling when in conversation.	-.22	**-.41**	.24
20. I often keep my emotions inside.	-.06	-.07	**-.80**
31. If I feel something, I prefer not to discuss it with others.	-.13	-.14	**-.75**
36. If I feel something, I rather not convey it to others.	-.04	-.11	**-.75**
44. I often know what I feel but choose not to reveal it outwardly.	.07	.13	**-.69**
48. If I feel something, I will convey it to others.	.21	.16	**.68**
9. It is hard for me to talk about my complex emotions.	-.09	-.37	**-.64**
45. If I feel something, it often comes pouring out of me.	.18	-.25	**.58**
30. People tell me I am good at expressing my emotions.	.27	.39	**.56**
2. I can express my emotions clearly to others.	.20	.50	**.51**
19. I am able to keep my emotions to myself if the timing to express them isn’t right.	.04	.44	**-.51**
73. I am more comfortable ‘talking around’ emotions I am feeling, rather than talking about them directly.	.00	-.16	**-.50**
40. Sometimes it is good to keep my emotions to myself.	.10	-.01	**-.48**
27. I am quick to act on my emotions.	.19	-.22	**.42**

*N* = 2,840. The largest loading for each item is highlighted in bold.

**Table 2:** Between-component correlations for 60-item MAS.

	Identifying	Processing	Expressing
Identifying	1	.24**	.24**
Processing	.24**	1	.22**
Expressing	.24**	.22**	1

*N* = 2,840;

*p < .05;

**p < .01

#### Hierarchical structure

The hierarchical structure of the one-component through three-component solutions was conducted using the procedure proposed by Goldberg [35]. First, a single component was specified in a PCA and then in two subsequent PCAs we specified two and three orthogonally rotated components. The component scores were saved for each solution. Next, correlations between component scores at adjacent levels were computed. The resulting hierarchical structure is displayed in Fig 1 (the hierarchical diagrams in this article were created in part by the Factor Diagrammer [Version 1.1b]) [36].

**Fig 3:**
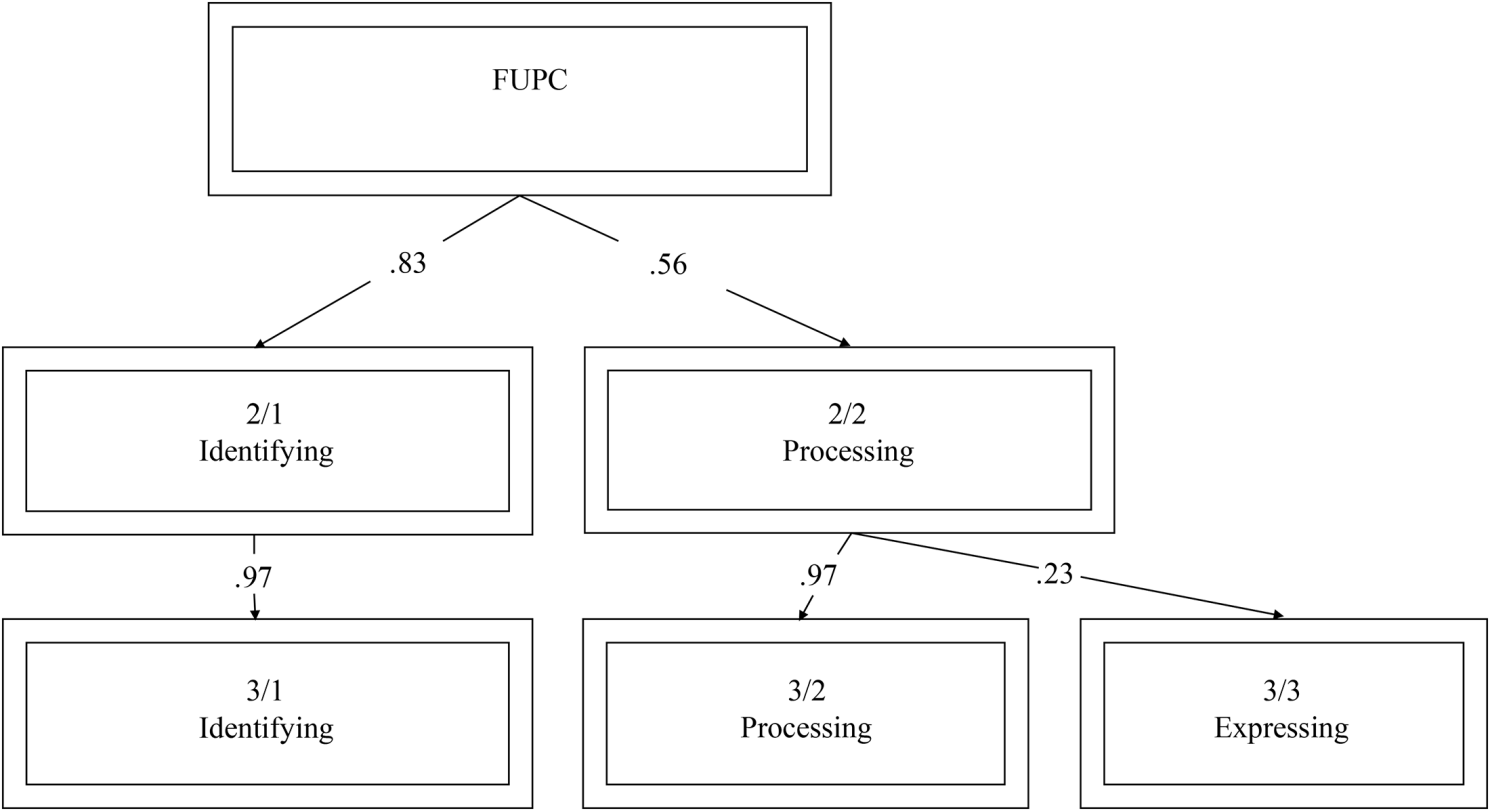
Varimax principal components derived from ratings for 60-items of the MAS. The figure begins (top box) with the First Unrotated Principal Component (FUPC) and displays the genesis of the derivation of the 3 components obtained. Text within each box indicates the label of the factor. Arabic numerals within boxes indicate the number of factors extracted for a given level (numerator) and the factor number within that level (denominator; e.g., 2/1 indicates the first component in a two-component solution). Arabic numerals within the arrow paths indicate the Pearson product-moment correlation between a component obtained early in the extraction and a later component. For example, when expanding from a two-component solution to a three-factor solution (rows 2 and 3), we see that Factor 2/2, “Processing” splits into two new factors, “Processing” (which correlates .97 with the parent component) and “Expressing” (which correlates .23 with the parent component).

Items that loaded highest on the one-component solution (FUPC) represented identifying and have a curiosity about understanding emotions, including “I often figure out where my emotions stem from”, “It is important for me to acknowledge my own true feelings”, and “I often look back at my life history to help inform my current emotional state and situation”. Items in the two-component solution appeared to represent “Identifying” and “Processing” dimensions of mentalized affectivity. Items that loaded highly on the “Identifying” dimensions were “I often look back at my life history to help inform my current emotional state and situation”, “I am curious about identifying my emotions”, and “I try to understand the complexity of my emotions”. This component remained virtually unchanged in the three-component solution. Items that loaded high on the “Processing” dimension were “I am good at distinguishing between different emotions that I feel”, “It is hard for me to manage my emotions”, “I can express my emotions clearly to others”, and “When I am filled with a negative emotion, I know how to handle it”, and “I am often confused about the emotions that I feel”. In the three-component solution, the “Processing” dimension split into two subcomponents that differentiated “Processing” affects from “Expressing” affects. Items that loaded highly on the “Expressing” dimension were, “I often keep my emotions inside”, “I often know what I feel but choose not to reveal it outwardly”, “If I feel something, I will convey it to others”, and “If I feel something, it often comes pouring out of me”. The hierarchical structure suggests that Processing delineates from Identifying, and Expressing delineates from Processing, which provides empirical support MA theory. The hierarchical structure is visually displayed in Fig 3.

#### Reliability

Cronbach’s alpha was calculated based on the scale scores from each of the three components. Reliability for the 24-item Identifying component was .93. Assessing Cronbach’s alpha for each item if it were removed did not go below .92. Reliability for the 23-item Processing component was .90. Assessing Cronbach’s alpha for each item if it were removed did not go below .90. Reliability for the 13-item Expressing component was .88. Assessing Cronbach’s alpha for each item if it were removed did not go below .86.

#### Convergent validity

Convergent validity was assessed with correlations between the three mentalized affectivity components and the EQ total and facet scores (cognitive empathy, social skills, and emotional reactivity). As empathy is defined as the ability to understand the thoughts and feelings of others and to predict others’ behavior based on that information (Baron-Cohen & Wheelwright, 2003), it would be expected that empathy would correlate with mentalized affectivity, which is concerned with understanding, processing, and communicating one’s own thoughts and feelings. As can be seen in Table 3, EQ total scores were correlated with Identifying, Processing, and Expressing. The same happened with Cognitive Empathy, with the highest correlation with Processing. Emotional Reactivity was most highly correlated with Identifying, and significantly correlated with Expressing. Social Skills was most highly correlated with Processing, and significantly correlated with Expressing.

**Table 3:** Convergent correlations with the Empathy Quotient.

	Mentalized Affectivity Components		
	Identifying	Processing	Expressing
EQ Total	.35**	.37**	.15*
Cognitive Empathy	.32**	.45**	.13*
Emotional Reactivity	.37**	-.03	.18**
Social Skills	.10	.45**	.20**

*N* = 267;

*p < .05;

**p < .01

### Part 2: The psychological correlates of the mentalized affectivity scale

#### Demographics

Independent samples t-tests showed that there was a significant difference between males and females for Identifying (*t*(2,084) = 8.04, p < .001), Processing (*t*(2,084) = -2.84, p < .001) and Expressing (*t*(1,790) = 3.59, p < .001). For Identifying, females (*M* = 5.33, *SD* = .82) scored higher than males (*M* = 5.02, *SD* = .93); for Processing, males (*M* = 4.67, *SD* = .82) scored higher than females (*M* = 4.56, *SD* = .83), and for Expressing, females (*M* = 4.58, *SD* = 1.03) scored higher than males ((*M* = 4.42, *SD* = .98). As seen in Table 4, age was positively correlated with Processing and Expressing, education was positively correlated with Processing, and income was negatively correlated with Identifying, but positively correlated with Processing and Expressing.

**Table 4:** External correlates of mentalized affectivity.

	Mentalized Affectivity Components		
	Identifying	Processing	Expressing
*Demographics*			
Age	-.05*	.15**	.11**
Education	.03	.08**	-.02
Income	-.08**	.07**	.06*
*Big Five*			
Openness	.23**	.20**	.09**
Conscientiousness	-.01	.30**	-.02
Extraversion	.05*	.18**	.41**
Agreeableness	.17**	.22**	.06**
Neuroticism	.23**	-.55**	.14**
*Well-Being*			
Life Satisfaction	-.03	.36**	.13**

*N* = 2,840;

*p < .05;

**p < .01

#### Personality and well-being

Correlations between mentalized affectivity, personality, and well-being are displayed in Table 4. Openness and Agreeableness was most positively correlated with Identifying and Processing, Conscientiousness was positively correlated with Processing, Extraversion was most positively correlated with Expressing, and Neuroticism was positively correlated with Identifying and Expressing, and negatively correlated with Processing. In terms of well-being, satisfaction with life scores was positively correlated with both Processing and Expressing.

#### Trauma

**Fig 4:**
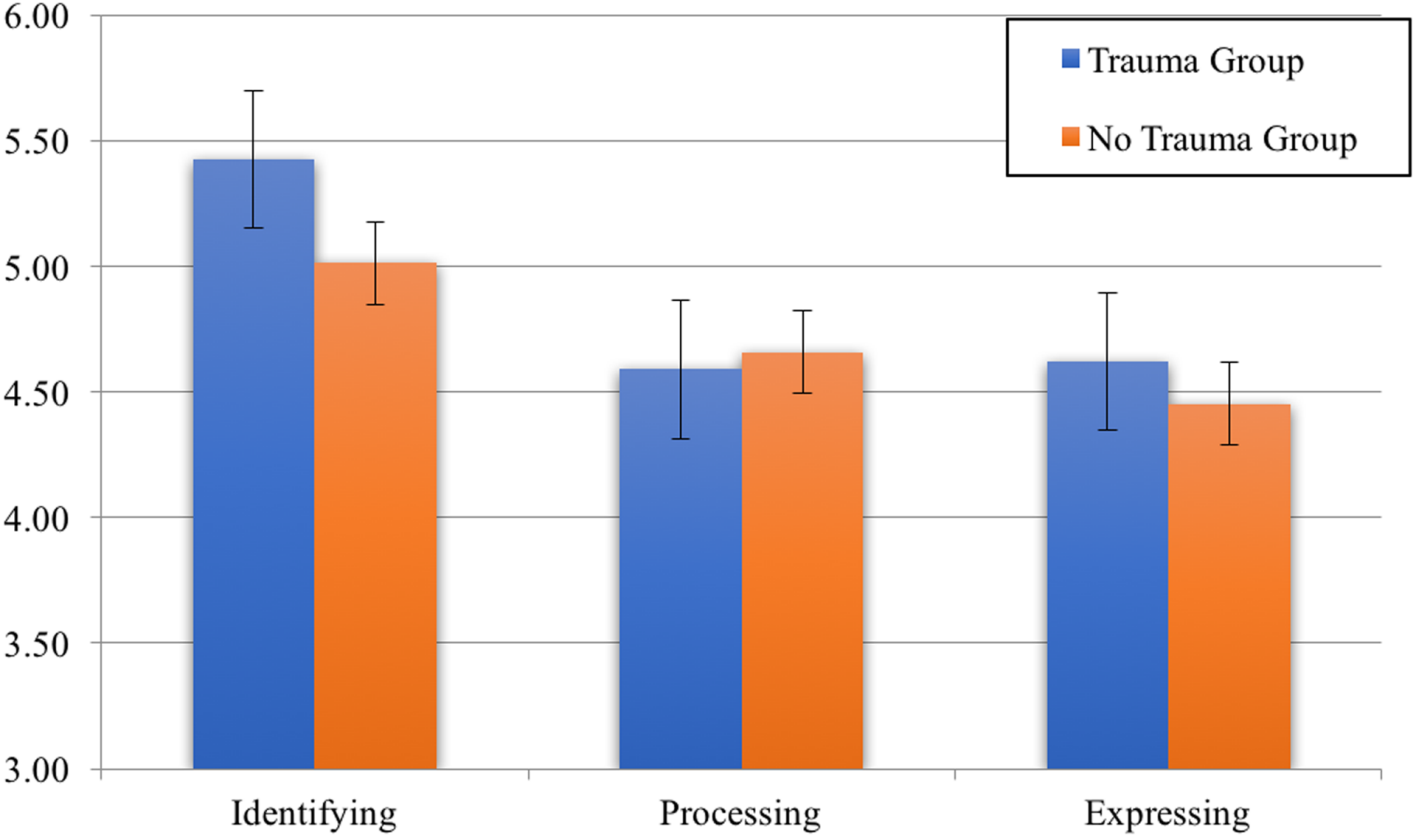
Mean differences between MA factors for trauma groups.

Independent samples t-tests showed that there was a significant difference between those who reported they experienced a recent (trauma group) and those who did not (no trauma group) for Identifying (*t*(1,790) = 10.08, p < .001) and Expressing (*t*(1,790) = 3.51, p < .001). For Identifying, the trauma group (*M* = 5.43, *SD* = .81) scored higher than the no trauma group (*M* = 5.01, *SD* = .91), and for Expressing the trauma group (*M* = 4.62, *SD* = 1.01) scored higher than the no trauma group (*M* = 4.45 *SD* = 1.01). There was no significant difference between the groups for Processing. Mean differences are displayed visually in Fig 4 (error bars indicate standard error). Mentalized affectivity components were correlated with trauma severity and the degree to which participants reported that they confided in others after the trauma. As can be seen in Table 5, trauma severity was positively correlated with Identifying, and confiding in others was most positively correlated with Expressing. There was no significant correlation between the MA components and the amount of time that had passed since the trauma.

**Table 5:** Mentalized affectivity correlates with recent trauma.

	Mentalized Affectivity Components		
	Identifying	Processing	Expressing
Severity of Trauma	.20**	-.06	.05
When Trauma Occurred	.00	.03	.00
Confided in Others	.13**	.20**	.32**

*N*s = 761 to 789;

*p < .05;

**p < .01

#### Linear regressions predicting well-being

We next performed hierarchical multiple regression models predicting life satisfaction. As seen in Table 6, in Model 1 we included only demographic variables (sex, age, education, and income), which accounted for 4% of the variance, with education and income demonstrating the largest associations with life satisfaction. In Model 2 we added the Big Five personality traits. This model accounted for 26% of the variance with Neuroticism most strongly associated (negatively) with life satisfaction). In Model 3 we added the three mentalized affectivity components. This model accounted for 28% of the variance with both Processing and Expressing being associated with life satisfaction. In Model 4 we added trauma variables. This model accounted for 32% of the variance. Both the severity of the trauma and the amount that participants reported confiding in others were significantly associated with life satisfaction. In this Model, Processing was most strongly associated with life satisfaction, behind Neuroticism, and confiding in others after the trauma.

**Table 6:** Multiple hierarchical linear regression models for predicting life satisfaction.

	*Model 1*			*Model 2*			*Model 3*			*Model 4*		
Variable	*B*	*(SE)*	*β*	*B*	*(SE)*	*β*	*B*	*(SE)*	*β*	*B*	*(SE)*	*Β*
(Constant)	3.54	.30		2.93	.47		3.34	.50		3.64	.78	
Sex	.15	.11	.05	.07	.10	.02	.08	.10	.03	.06	.10	.02
Age	-.01	.00	-.05	-.02	.00	-.15**	-.02	.00	-.17**	-.02	.00	-.15**
Education	.14	.05	.10*	.10	.05	.07*	.09	.05	.06	.08	.05	.05
Income	.04	.01	.16**	.03	.01	.10*	.03	.01	.11*	.03	.01	.11*
Openness				.04	.05	.03	.01	.05	.01	.04	.05	.02
Conscientiousness				.19	.04	.18**	.15	.04	.15**	.15	.04	.14**
Extraversion				.18	.03	.20**	.14	.03	.15**	.12	.03	.14**
Agreeableness				.18	.04	.16**	.16	.04	.14**	.16	.04	.14**
Neuroticism				-.27	.04	-.27**	-.22	.04	-.22**	-.18	.04	-.18**
Identifying							-.01	.06	-.01	.01	.06	.00
Processing							.23	.06	.17**	.22	.06	.16**
Expressing							.11	-.05	.08*	.08	-.05	.06
Recent Trauma (0 = no, 1 = yes)										-.35	.61	-.02
Trauma Severity										-.13	.03	-.17**
When trauma occurred										.00	.00	.04
Confided in others										.07	.03	.10*

*N* = 693. For Model 1 the *R*^2^ is .04 and the *F* for the change in *R*^2^ is 6.81 (*p* < .001). For Model 2 the *R*^2^ is .26 and the *F* for the change in *R*^2^ is 40.62 (*p* < .001). For Model 3 the *R*^2^ is .28 and the *F* for the change in *R*^2^ is 7.54 (*p* < .001). For Model 4 the *R*^2^ is .32 and the *F* for the change in *R*^2^ is 8.34 (*p* < .001).

*p < .05;

**p < .01.

### Part 3: Links to psychological disorders and treatment

#### 18 psychological disorders vs controls

We next examined how MA components differed across clinical diagnoses. Table 7 reports the sample characteristics across clinical diagnoses. The control group consisted of only those who denied having had been diagnosed with any of the 18 disorders presented to them. Each of the clinical groups included participants who indicated they had been diagnosed with the respective disorder, including those who indicated multiple diagnoses (i.e. comorbidities). We only included groups in the analysis who had an *N* of 10 or above.

**Table 7:** Sample characteristics of clinical diagnoses.

Diagnosis	*N*	*N* (not comorbid)	%
Control Group	1,448	—	51.0
*Anxiety Disorders*			
GAD	230	39	8.1
OCD	43	4	1.5
Panic Disorder	68	10	2.4
PTSD	73	15	2.6
Social Anxiety Disorder	125	19	4.4
*Eating Disorders*			
Anorexia	18	1	0.6
Bulimia	16	2	0.6
*Mood Disorders*			
Bipolar	44	5	1.5
Depression	412	144	14.5
SAD	33	3	1.2
*Neurodevelopmental Disorders*			
ADHD	125	47	4.4
Autism	29	13	1.4
*Other*			
Alexithymia	1	0	0
Epilepsy	10	5	0.4
Synesthesia	26	3	0.9
*Personality Disorders*			
Borderline	24	3	0.8
Narcissistic	3	0	0.1
*Psychotic Disorders*			
Schizophrenia	4	0	0.1

Considering some of the clinical groups were relatively small, in order to generate the most robust conclusions possible, we first performed a comparison of a clinical group comprising of all those who indicated a diagnosis vs controls who did not indicated a diagnosis.

We performed ANCOVAs controlling for sex and age. For Identifying, the clinical group (*N* = 620; *M* = 5.44; *SD* = .80) scored higher than the control group (*N* = 1416; *M* = 5.08; *SD* = .90) [*F* = 57.79, p < .001]. For Processing, the clinical group (*M* = 4.38; *SD* = .87) scored lower than the control group (*M* = 4.70; SD = .78)[*F* = 67.80, *p* < .001]. The difference between the groups for Expressing was not significant.

Continuing with comparisons of larger groups, we then performed group comparisons comparing controls to anxiety disorders (GAD, OCD, Panic Disorder, PTSD, and Social Anxiety Disorder), eating disorders (Anorexia and Bulimia), Mood Disorders (Bipolar, Depression, and SAD), Neurodevelopmental Disorders (ADHD and Autism), and Personality Disorders (Borderline Personality Disorder and Narcissistic Personality Disorders). Table 8 reports the results from each of the ANCOVAs. Because sex and age were included as covariates, and not all participants indicated their sex and age, as can be seen, some of the *N*s indicated in Table 9 for each clinical group are smaller than their respective *N*s reported in Table 7. Specifically, the *M* and *SD* are reported for the control group and each clinical group, and the *F* and *p* values from the ANCOVAs are reported for each respective clinical group. The Anxiety Disorder group scored higher on Identifying and lower on Processing than controls; the Eating Disorder group had no significant differences than controls; the Mood Disorder group score higher on Identifying and lower on Processing than controls; the Neurodevelopmental Disorder group scored higher on Identifying, lower on Processing, and higher on Expressing than controls (the differences in expressing was driven by the participants with ADHD and not autism—a more detailed analysis is described below); and the Personality Disorder group, made up primarily of Borderline Personality Disorders, scored higher on Identifying and lower on Processing than controls.

**Table 8:** MA mean comparisons across clinical diagnoses.

		Identifying				Processing				Expressing			
Diagnosis	*N*	*M*	*SD*	*F*	*p*	*M*	*SD*	*F*	*p*	*M*	*SD*	*F*	*p*
Control Group (no diagnosis)	1416	5.09	.90	-	-	4.70	.78	-	-	4.48	.99	-	-
*Anxiety Disorders*	340	5.52	.80	51.71	.000	4.32	.88	52.91	.000	4,59	1.07	2.47	.116
*Eating Disorders*	29	5.33	.71	0.46	.497	4.43	.79	1.90	.168	4.46	1.25	.36	.552
*Mood Disorders*	412	5.48	.80	51.36	.000	4.28	.86	82.02	.000	4.50	1.00	.396	.529
*Neurodevelopmental Disorders*	140	5.43	.82	17.77	.000	4.49	.93	7.35	.007	4.67	1.10	5.27	.022
*Personality Disorders*	26	5.63	.86	7.75	.005	4.32	1.07	5.25	.022	4.71	1.11	1.14	2.86

**Table 9:** MA mean comparisons across clinical diagnoses.

		Identifying				Processing				Expressing			
Diagnosis	*N*	*M*	*SD*	*F*	*p*	*M*	*SD*	*F*	*p*	*M*	*SD*	*F*	*p*
Control Group (no diagnosis)	1416	5.09	.90	-	-	4.70	.78	-	-	4.48	.99	-	-
*Anxiety Disorders*													
GAD	217	5.55	.79	37.93	.000	4.30	.94	39.18	.000	4.68	1.09	6.18	.013
OCD	42	5.33	.83	2.92	.088	3.95	.85	36.49	.000	4.22	.75	2.45	.117
Panic Disorder	67	5.47	.84	7.95	.005	4.25	.83	19.13	.000	4.58	1.13	.37	.545
PTSD	66	5.75	.74	25.26	.000	4.30	1.00	16.36	.000	4.78	1.00	2.82	.093
Social Anxiety Disorder	118	5.57	.72	26.65	.000	4.25	.83	28.17	.000	4.22	.97	6.79	.009
*Eating Disorders*													
Anorexia	18	5.27	.54	.03	.854	4.32	.77	2.93	.087	4.39	1.09	.25	.614
Bulimia	16	5.27	.84	.06	.799	4.73	.78	.18	.675	4.80	1.34	1.28	.258
*Mood Disorders*													
Bipolar	41	5.59	.74	9.90	.002	4.23	.86	13.42	.000	4.59	1.07	.24	.625
Depression	393	5.48	.78	49.60	.000	4.28	.86	83.04	.000	4.54	1.03	.34	.558
SAD	32	5.36	.76	1.54	.214	4.41	.98	4.06	.044	4.70	.92	.84	.358
*Neurodevelopmental Disorders*													
ADHD	120	5.50	.81	20.69	.000	4.60	.88	1.32	.251	4.74	1.07	7.38	.007
Autism	26	5.25	.84	1.07	.300	3.98	.99	21.15	.000	4.40	1.17	.08	.780
*Other*													
Alexithymia		-	-	-	-	-	-	-	-	-	-	-	-
Epilepsy	10	5.22	.99	.14	.705	4.67	.95	.04	.851	4.02	1.18	2.62	.105
Synesthesia	25	5.46	.99	2.18	.140	4.42	1.06	2.34	.126	4.60	.98	.18	.672
*Personality Disorders*													
Borderline	23	5.63	.82	6.17	.013	4.29	.99	5.22	.022	4.75	1.17	1.50	.221
Narcissistic		-	-	-	-	-	-	-	-	-	-	-	-
*Psychotic Disorder*													
Schizophrenia		-	-	-	-	-	-	-	-	-	-	-	-

Because each of the diagnoses that comprised the previous diagnostic groups can be characteristically different from each other (e.g. ADHD and Autism in Neurodevelopmental Disorders), we therefore next performed more detailed analyses by performing ANCOVAs for each of the clinical groups compared to controls. There was a total of 18 ANCOVAs performed reported in Table 9. As in Tables 8 and 9 the *M* and *SD* are reported for the control group and each clinical group, and the *F* and *p* values from the ANCOVAs are reported for each respective clinical group.

**Fig 5:**
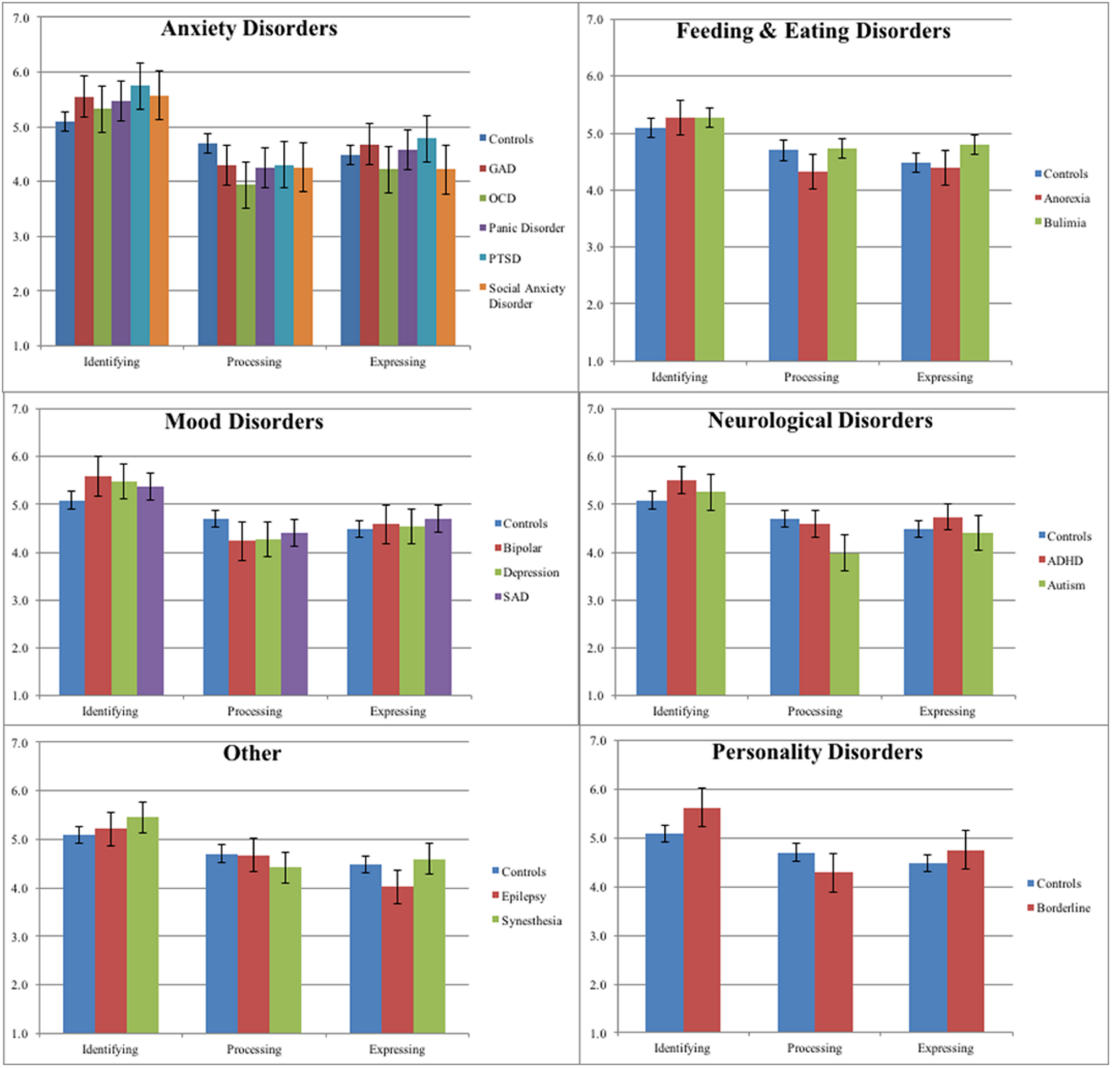
Mean differences of MA components across clinical diagnoses.

All of the anxiety disorders showed this pattern (except for OCD which did not have a significant difference from controls for Identifying). The GAD and social anxiety disorder groups both scored higher on Expressing than controls. For eating disorders, there was no significant difference between any of the MA components, suggesting the mean differences between eating disorders and controls were accounted for by sex differences in the groups. Both bipolar and depression groups scored higher on Identifying and lower on Processing than controls. The seasonal affective disorder (SAD) group scored significantly lower on Processing but not Identifying. There were no significant differences between the mood disorder groups and control groups on Expressing. Those with ADHD scored higher on Identifying than controls and those with autism scored lower on Processing than controls. The ADHD group scored higher on Expressing than the control group. In terms of other groups that were unclassified, epilepsy and synesthesia groups had no significant differences than controls for the MA components. The borderline group scored higher on Identifying and lower on Processing than controls. Mean differences are visually displayed in Fig 5. Overall, these comparisons show that the components of mentalized affectivity are linked to psychopathology with a general pattern of high Identifying and low Processing.

#### Psychological treatment

**Fig 6:**
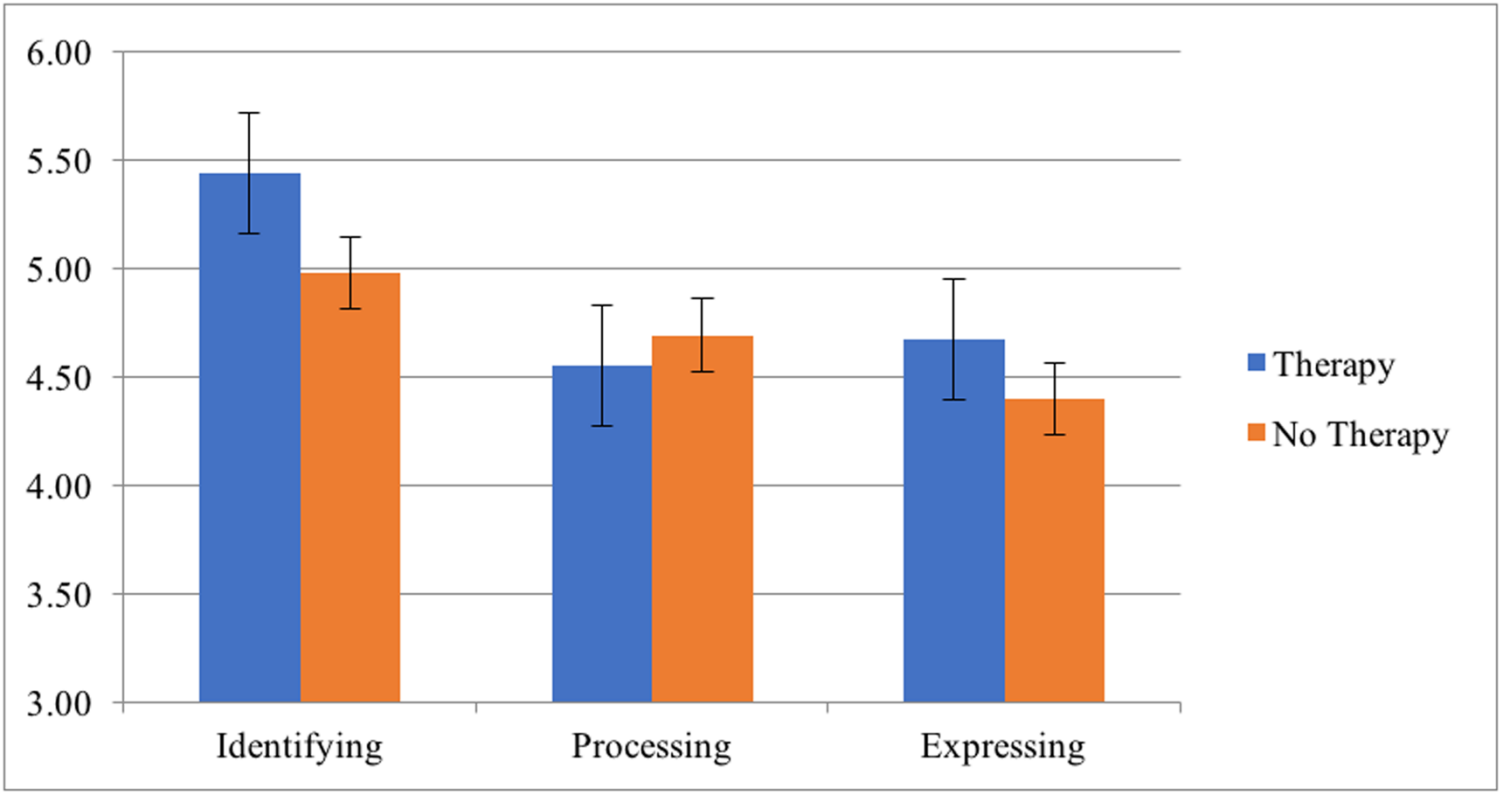
Differences in MA scores across therapy and no therapy groups.

Sample characteristics and mean differences in MA components for each of the psychological treatment variables are displayed in Table 10. We first performed an ANCOVA comparing those in therapy compared to those not currently in therapy. The currently in therapy group scored higher than the not currently in therapy group on Identifying (*F*(1, 1435) = 36.80, *p* < .001), lower on Processing (*F*(1, 1435) = 28.71, *p* < .001), and higher on Expressing (*F*(1, 1435) = 11.23, *p* < .01). We next performed ANCOVAs between therapy group (those who are both currently and have had prior therapy but not currently in it) and no therapy group (no current or prior therapy). The results replicated the analysis of those currently in therapy: The therapy group scored higher on the no therapy group on Identifying (*F*(1, 2058) = 127.48, *p* < .001), lower on Processing (*F*(1, 2058) = 21.32, *p* < .001), and higher on Expressing (*F*(1, 2058) = 20.72, *p* < .001). A visual mean comparison of the trauma group vs. no trauma group is displayed in Fig 6.

**Table 10:** Sample characteristics and MA mean differences across therapeutic treatment variables.

					Identifying		Processing		Expressing	
Variable	*N*	%	N (only)	% (only)	*M*	*SD*	*M*	*SD*	*M*	*SD*
*Currently in therapy*										
Yes	247	12.2	—	—	5.63	0.81	4.33	0.87	4.73	1.10
No	1,753	86.9	—	—	5.15	0.88	4.63	0.80	4.45	0.98
Rather not say	17	0.8	—	—	—	—	—	—	—	—
*Previously or currently in therapy*										
Yes	1263	44.8	—	—	5.44	0.84	4.55	0.85	4.67	1.01
No	1557	55.2	—	—	4.98	0.90	4.69	0.77	4.40	0.99
*Years in therapy*										
0.5 to 1 year	598	47.3	—	—	5.31	0.83	4.59	0.83	4.60	0.99
1.5 to 2 years	223	17.7	—	—	5.46	0.76	4.56	0.78	4.70	0.98
2.5 to 3 years	115	9.1	—	—	5.46	0.90	4.44	0.92	4.58	0.96
3.5 to 4 years	63	5.0	—	—	5.69	0.94	4.63	0.97	4.90	1.16
4.5 to 5 years	73	5.8	—	—	5.62	0.79	4.52	0.82	4.85	1.04
More than 5 years	191	15.1	—	—	5.64	0.82	4.47	0.90	4.75	1.06
*Type of therapy*										
I do not know	257	9	—	—	—	—	—	—	—	—
I have never been in therapy	1557	55.2	—	—	5.09	0.89	4.64	0.80	4.45	1.00
Cognitive behavioral therapy	348	12.3	155	6.1	5.50	0.80	4.47	0.95	4.75	1.03
Couples therapy	90	3.2	39	1.5	4.85	0.85	4.63	0.96	4.68	1.03
Creative arts therapy	30	1.1	3	.1	5.90	0.21	4.48	0.18	4.81	0.60
Dialectical behavior therapy	42	1.5	3	.1	5.29	0.94	4.76	0.77	5.23	0.33
Exposure therapy	22	0.8	1	.0	4.42	—	3.30	—	4.77	—
Family therapy	101	3.6	45	1.8	5.27	0.73	4.70	0.94	4.58	0.97
Group therapy	93	3.3	12	.5	5.63	0.77	4.80	1.01	4.91	1.21
Mindfulness-based therapy	141	5	32	1.3	5.73	0.66	4.73	0.74	4.65	0.81
Psychoanalysis	102	3.6	35	1.4	5.42	0.47	4.51	0.86	4.85	1.05
Psychotherapy	329	11.6	162	6.3	5.39	0.84	4.56	0.83	4.61	1.01

#### Years in treatment

**Fig 7:**
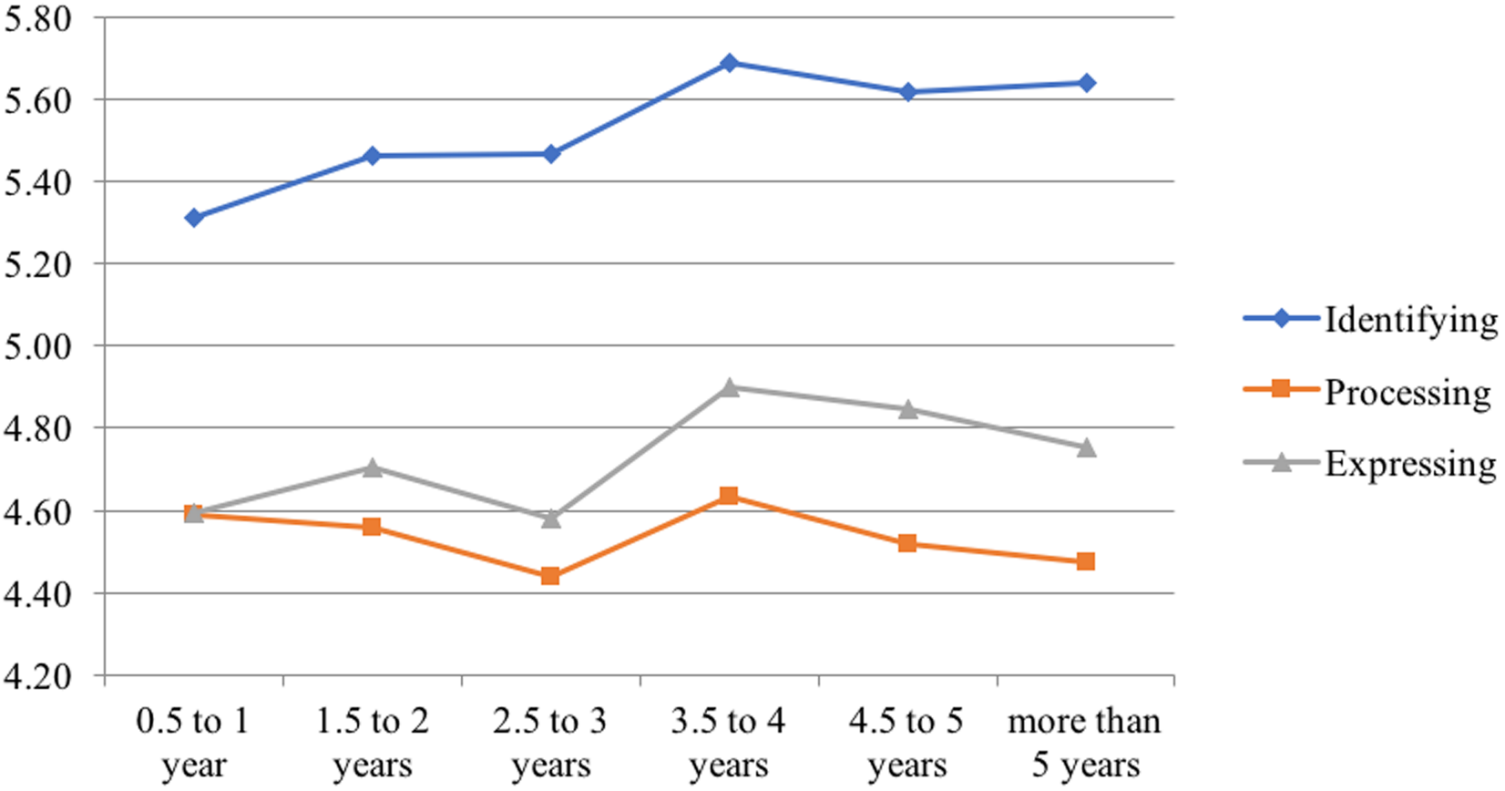
Mean scores on MA components across years in therapy.

We next examined the links between years in treatment and mentalized affectivity. Responses ranged from .5 to 59 years in therapy. Zero-order correlations showed that years in therapy was positively associated with Identifying (*r* = .09, *p* < .001) and expressing (*r* = .06, *p* < .05). There was no significant association between years in therapy and Processing (*r* = —.04, *p* = .14). However, the responses to years in therapy were skewed as the majority of responses indicated that subjects had between .5 and 5 years of therapy. Therefore, to get a better indication of the links between years in therapy and mentalized affectivity, we separated years in therapy into 6 groups (.5 to 1 year in therapy; 1.5 to 2 years in therapy; 2.5 to 3 years in therapy, 3.5 to 4 years in therapy, 4.5 to 5 years in therapy, and more than five years in therapy). We then performed ANOVAs with the years in therapy groups as the IV and each of the MA components as the DV. Results showed that there was a significant effect for Identifying (*F*(5, 1257) = 6.99, *p* < .001) and Expressing (*F*(5, 1257) = 2.26, *p* = .46). Post-hoc Tukey tests revealed that for Identifying, the .5 to 1 year of therapy group scored significantly lower than the 3.5 to 4 years in therapy group, the 4.5 to 5 years in therapy group, and the more than 5 years in therapy group. Post-hoc Tukey tests revealed no significant differences between groups for Processing or Expressing. Mean MA scores between groups are displayed visually in Fig 7.

#### Types of psychological treatment

**Fig 8:**
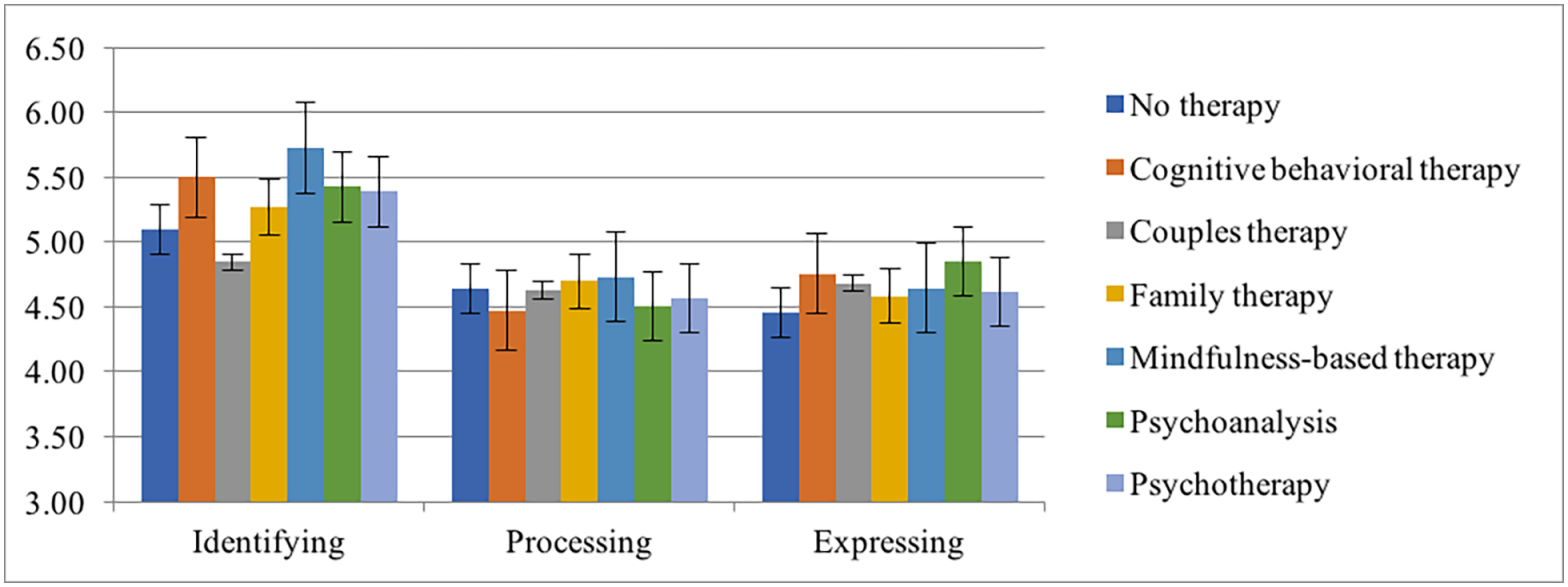
MA scores across different therapy treatments.

We next examined mean differences between MA scores and reported treatment modalities. The control group consisted of participants who indicated they had no history of psychological treatment and those in each treatment group consisted of participants who indicated that they only had the type of treatment in question (i.e. participants who indicated they had multiple treatment modalities were not included in the treatment groups). As several of the groups had small *N*s we included No Therapy, Cognitive Behavioral Therapy, Couples Therapy, Family Therapy, Mindfulness-based Therapy, Psychoanalyses, and Psychotherapy groups in the analysis. We performed an ANOVA with treatment groups as the IV and MA component scores as the DV. There was a significant effect for all three MA components: *F*(6, 2506) = 9.07, *p* < .001) for Identifying, *F*(6, 2506) = 2.53, *p* < .05) for Processing, and *F*(6, 2506) = 3.20, *p* <. 01) for Expressing. Post-hoc Tukey tests showed that the CBT, Mindfulness-based Therapy, and Psychotherapy treatment groups scored higher on Identifying than controls for Identifying. The Mindfulness-based Therapy group scored higher than Couples Therapy for Identifying. The CBT group scored significant lower on Processing than controls. The difference between the CBT group and control group for Expressing approached significance (*p* = .50). Mean differences are displayed visually in Fig 8.

## Discussion

### Summary

The present study provides a general and specific overview of the theory and assessment of mentalized affectivity. In part 1, consistent with our initial hypothesis, results showed three robust and transparent components underlying mentalized affectivity: Identifying, Processing, and Expressing. Hierarchical modeling showed how Processing delineated from Identifying, and Expressing delineated from Processing. The MAS is the first emotion regulation measure that captures all three of these concepts in a single measure and is the first measure to bring emotion regulation and mentalization together. The results also showed that the measure had high reliability and validity.

In part 2 of the results, we showed how the three MA factors were associated with demographics, personality traits, well-being, and trauma histories. Linear regressions showed that when controlling for all other variables (demographics, personality, and trauma) that Processing was positively correlated with life satisfaction. This provides initial evidence that Processing is a key element to focus on in psychological treatment. Personality correlation confirmed and contrasted our initial predictions. For example, we did not anticipate that Neuroticism would be positively correlated to Identifying and negatively correlated with life satisfaction.

In part 3 of the results, we demonstrated how MA factors were linked to psychological diagnoses and psychological treatment. Results showed a trend in which psychological disorders displayed a pattern of high Identifying and low Processing scores. Although we had accurately predicted that low Processing scores would be feature of psychological disorders, we did not anticipate that high Identifying scores would be a feature as well. Taking into consideration that the high Identifying scores were present for anxiety disorders, mood disorders, and personality disorders, and that Identifying was positively correlated with Neuroticism, future research should explore the etiology and development of personality traits, emotion regulation, and clinical diagnoses and to generate a better understanding of causation. It may be that those who struggle with anxiety, mood, and relationships use Identifying (participating the aspect of idetnfiyng where the emotions stem from) as an adaptive strategy to better understand themselves and their condition in an attempt (whether successful or not) to improve their well-being.

The results from histories of psychological treatment were less robust than the results from clinical diagnoses. These results indicated that Identifying emotions were higher for those with 3.5 or more years of therapy. Comparisons between treatment modalities showed that those subjects who had participated in CBT, Mindfulness-based treatments, and Psychotherapy scored higher on Identifying than those who had not engaged in psychological treatment.

### Implications for emotion regulation and mentalization

The newly developed MAS can be useful for researchers and clinicians studying emotion regulation and mentalization. In the introduction, we outlined how prior measures of emotion regulation have captured aspects of Identifying, Processing, and Expression, but that none of these prior measures captures all three in a single measure. Further, several popular measures in the past focused on deficits and dysfunction of emotion regulation, rather than a broader spectrum of emotion regulation applied to typical functioning. Our results have addressed this prior limitation in theory and both measurement application. Those interested in a multi-dimensional approach to emotion regulation may find the MAS useful. Development, cognitive, personality, social psychologists, along with neuroscientists and Big Data scientists may find this measure particularly useful either as a primary or supplement focus in their studies.

The measure may also be relevant for those studying mentalization and reflective functioning. Prior measures of reflective functioning focus on one’s cognitive and affective ability to understand the thoughts and feelings of oneself and others. The MAS extends those measures not only by including aspects of processing emotions and expressing them, but also by highlighting the ability to link emotions to prior and present contexts. This is a fundamental theme within the theory of mentalization, which has not been explicitly incorporated in the current self-report measures within reflective functioning. Indeed, measures on mentalization have been criticized regarding their validity, usage, the time and expense required, and the broadness of its conceptualization [37]. Critiques of mentalization assessments may find that the MAS addressed some of these concerns both conceptually and psychometrically.

Taken together, the MAS is an intriguing addition to the literature of pre-existing measures on the topic. Indeed, given the numerous emotion regulation and mentalization measures that exist, researchers and clinicians may choose to determine which measure to use in their work based on the research or clinical situation and the dimensions that they would like to measure. For example, the DERS has several dimensions that capture maladaptive behavior that the MAS does not, including their Nonacceptance of emotional responses (Nonacceptance), Difficulties in Engaging in Goal-Directed (Goals), and Impulse of Control Difficulties, dimensions (Impulse). And rather than just measuring unipolar direction of mentalization, the RFQ makes the distinction between certainty and uncertainty of mental states. The FREE measures expression and suppression of emotions given specific positive and negative contexts. In total, the literature on emotional regulation and mentalization has a rich source of measures from which researchers and clinicians can choose from based on their research and therapeutic goals.

### Implications for clinical psychology and psychiatry

Our results demonstrated a trend that those with psychological disorders scored high in Identifying emotions and low in Processing, a result which may be useful in the context of supplementing diagnosis and assessment. There has been a growing movement to understand psychopathology in terms of transdiagnostic criteria rather than classification systems employed by manuals such as the DSM-5 or ICD-10 [38]. Indeed, several of these proposals suggest a bi-factorial model of psychopathology [39–40]. Part of this movement is in response to the idea that comorbidities are the “rule” rather than the exception [38]. Our results, if replicated in different clinical contexts and using different study designs (e.g. longitudinal data), may help to better understand these transdiagnostic models and comorbidities.

In terms of treatment, although these results are based on retrospective accounts and causation cannot be inferred, it is interesting that Identifying was linked both to years in treatment as well as modalities in psychological treatment, but Processing (not Identifying) was linked to well-being across the general population. Various modalities of treatment, including CBT and psychotherapy have a long history of enabling patients to identify emotions and understand their meaning within present and prior contexts. Expressing emotions and being able to communicate them is also a valued concept. However, Identifying was not linked to life-satisfaction scores, and Expressing was not a significant predictor after controlling for prior trauma variables. This suggests that psychological treatments may want to focus more on processing emotions than only identifying them. There may be false beliefs, where the goal of treatment is simply to identify emotions and understand their meaning. However, these results suggest that without the capacity to modulate and refine them, well-being will not be increased.

The MAS is intriguing as a clinical tool: as a way to better understand what kinds of patients have what kinds of problems with using emotions. Our measure provides support for Jurist’s (in press) discussion of interventions to cultivate mentalized affectivity in patients, based upon specifying problems with using emotions. A final thought to ponder is whether every treatment—regardless of orientation—aims to improve mentalized affectivity. And further, whether targeting mentalized affectivity could be just as effective in short-term as in long-term treatment [41].

### Limitations and future directions

The main limitation of the present study is that it relied on self-reports and retrospective accounts. Therefore, causation cannot be inferred from the results. To overcome this limitation, future research should administer this assessment longitudinally in clinic settings such as hospitals and community clinics. This will this advance understanding regarding the ways in which mentalized affectivity plays a role in therapeutic growth and development, but it can also provide valuable metrics for psychologists and psychiatrists to gage the progress of their patients throughout treatment.

Social, cognitive and affective neuroscience have begun to explore aspects of emotion regulation neurobiologically [42–44]. For example, a recent meta-analysis showed increases in activity in bilateral amygdala/ parahippocampal gyrus during “upregulated states” but decreased in “downregulated” states [45]. Neuroscience has also showed interesting patterns regarding mentalization through imaging studies [46–47]. Neuroscience may find the MAS a complementary measure of trait emotion regulation and mentalization.

An important limitation of the present research is the method used to obtain information about clinical diagnoses. Because of the large sample size, we were unable to employ additional checks for diagnoses such as follow-up phone interviews about diagnoses. Indeed, we obtained diagnostic information along similar standards used in previous online studies that have examined clinical diagnoses in large samples [48]. More detailed analyses regarding MA components and clinical diagnoses including etiology could be optimized in the future by conducting research in clinic settings.

## Conclusion

In this paper we advanced prior theory and research by introducing a new model and assessment of emotion regulation. We used a large sample to show how three distinct aspects of mentalized affectivity (identifying, processing, and expressing emotions) are linked to personality traits, well-being, trauma, diagnoses of psychological disorders, and histories of psychological treatment. The mentalized affectivity scale (MAS) is a multi-dimensional and reliable tool that can be useful in future research in both clinical and non-clinical settings and within many areas of areas of psychology, psychiatry, and neuroscience.

## Supporting information

S1 Fig Scree Plot from PCA. (TIF)

S1 Table Component Loadings for the initial 76-items of the MAS. (PDF)

S1 Dataset (SAV)
