# Supplementary figures and images for: Mentalized affectivity: A new model and assessment of emotion regulation
Source: PLoS One. 2017 Oct 18;12(10):e0185264. doi: 10.1371/journal.pone.0185264 (PMC5646776; doi:10.1371/journal.pone.0185264)

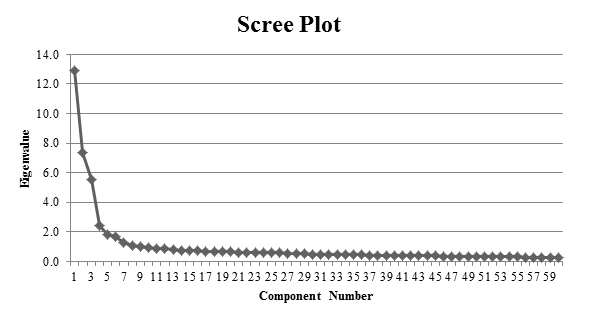

Supplement: S1 Fig — (TIF) [file pone.0185264.s001.tif]
