# Supplementary material for: Mentalized affectivity: A new model and assessment of emotion regulation
Source: PLoS One. 2017 Oct 18;12(10):e0185264. doi: 10.1371/journal.pone.0185264 (PMC5646776; doi:10.1371/journal.pone.0185264)
Supplement: S1 Table — (PDF) [file pone.0185264.s002.pdf]

**S1 Table. Component Loadings for the initial 76-items of the MAS.**			
	Principal Component		
	I	II	III
50. I often look back at my life history to help inform my current emotional state and situation.	**.76**	.05	-.02
56. I am curious about identifying my emotions.	**.73**	.03	-.08
60. I try to put effort into identifying my emotions.	**.73**	.07	-.03
3. I often think about my past experiences to help me understand emotions that I feel in the present.	**.73**	-.05	-.05
47. I try to understand the complexity of my emotions.	**.72**	.11	-.05
70. It's important to understand the major life events that have had an impact on my behavior.	**.72**	.06	-.03
62. Knowing about the childhood experiences of others helps me to understand their emotions.	**.71**	.17	-.01
67. It is helpful to think about how my emotions stem from family dynamics.	**.69**	.08	-.05
1. I often think about how the emotions that I feel stem from earlier life experiences (e.g. family dynamics during childhood).	**.67**	-.16	-.05
27. Knowing about my childhood experiences helps to put my present emotions within a larger context.	**.67**	.07	-.01
61. I can pinpoint childhood experiences that influence the way that I often think and feel.	**.66**	.10	.01
43. I try to understand how other people's behavior is influenced by their past history.	**.65**	.23	.01
51. I am curious about identifying others' emotions.	**.65**	.17	-.07
8. I am interested in learning about why I feel certain emotions more frequently than others.	**.64**	-.12	-.12
48. It is important for me to acknowledge my own true feelings.	**.64**	.18	-.19
66. I can see how prior relationships influence the relationships that I have now.	**.64**	.18	-.04
64. Thinking about other people's emotional experiences helps me to think about my own.	**.61**	.13	-.15
41. It helps me to know the reasons behind why I feel the way that I do.	**.60**	.07	.00
32. I try to understand how the major life evens of other people influences how they feel.	**.60**	.26	.04
17. I can see how prior relationships influence my current emotions.	**.60**	.11	-.03

23. Understanding my emotional experience is an ongoing process.	**.59**	-.13	-.06
10. I try to understand the complexity of other people's emotions.	**.58**	.16	-.06
49. I often figure out where my emotions stem from.	**.55**	.43	-.08
69. I rarely think about the reasons behind why I am feeling a certain way.	**-.54**	-.03	.10
42. I am aware of recurrent patterns to my emotions.	**.53**	.23	-.03
2. I put effort into managing my emotions.	**.50**	.02	.11
15. I use tools I have learned to help when I am in difficult emotional situations.	**.45**	.24	.01
68. I am open to other people's view of me because it helps me to better understand myself.	**.42**	.13	-.04
52. I am open to what others say about me to help me know what I am feeling.	**.41**	.11	-.01
72. It can help me to know how I feel, if I know how others feel.	.38	-.09	-.04
40. Knowing another's thoughts about me can be helpful to identify my emotions.	.36	-.03	.02
58. I know what environmental triggers will evoke certain emotions in me (such as anger).	.33	.32	.02
16. I can easily predict how I will react to someone based on my previous life history.	.31	.27	.04
14. It's difficult to understand how past life events have influenced my behavior.	-.31	-.30	.02
24. I am often confused about the emotions that I feel.	.15	**-.68**	.09
55. I am good at distinguishing between different emotions that I feel.	.27	**.67**	-.01
26. It is hard for me to manage my emotions.	.20	**-.66**	-.20
74. I can quickly identify my emotions without having to think too much about it.	.09	**.65**	-.17
21. When I am filled with a negative emotion, I know how to handle it.	-.03	**.63**	.21
76. I can tell if I am feeling a combination of emotions at the same time.	.34	**.61**	-.07
75. I am able to understand my emotions within the context of my surroundings.	.28	**.60**	-.06
22. I often know the reasons why I feel the emotions I do.	.19	**.59**	-.06
35. I am good at controlling my emotions.	-.13	**.58**	.44

12. I can express my emotions clearly to others.	.17	**.57**	-.44
36. When I express my emotions to others, it is usually jumbled.	.04	**-.56**	.30
38. When I am filled with a positive emotion, I know how to keep the feeling going.	.05	**.54**	-.06
46. It takes me a while to know how I am really feeling.	.12	**-.54**	.29
53. People get confused when I try to express my emotions.	.06	**-.53**	.29
25. I am able to adjust my emotions to be more precise.	.11	**.53**	.16
13. I am good at understanding other people's complex emotions.	.37	**.52**	-.17
29. It's easy for me to understand the thoughts and feelings of others.	.34	**.52**	-.12
28. It is easy for me to notice when I am feeling different emotions at the same time.	.37	**.51**	-.03
18. I can still think rationally even if my emotions are complex.	.03	**.51**	.33
39. I am good at controlling emotions that I do not want to feel.	-.13	**.50**	.31
33. I can easily label "basic emotions" (fear, anger, sadness joy and surprise) that I feel.	.26	**.50**	-.04
65. I can easily label "basic emotions" that others feel.	.38	**.49**	-.05
37. It is easy for me to notice when others are feeling different emotions at the same time.	.34	**.48**	-.03
34. I am good at increasing emotions that I want to feel more.	.12	**.48**	-.07
9. I am often confused about the emotions that others feel.	-.05	**-.48**	.21
71. I am not aware of the emotions I'm feeling when in conversation.	-.19	**-.46**	.19
11. When talking with someone, I am aware of both my emotions and theirs.	.42	**.45**	-.11
57. If a feeling makes me feel uncomfortable, I can easily get rid of it.	-.19	**.45**	.23
7. I am aware of the emotions I feel most frequently.	.34	.36	-.17
31. I often keep my emotions inside.	-.04	.16	**-.78**
45. If I feel something, I prefer not to discuss it with others.	.11	.23	**-.73**
5. If I feel something, I rather not convey it to others.	.02	.20	**-.72**
59. I often know what I feel but choose not to reveal it outwardly.	-.07	-.06	**-.69**
63. If I feel something, I will convey it to others.	-.20	-.24	**.65**

6. If I feel something, it often comes pouring out of me.	-.19	.16	**.61**
20. It is hard for me to talk about my complex emotions.	.06	.44	**-.59**
30. I am able to keep my emotions to myself if the timing to express them isn't right.	-.03	-.37	**-.56**
19. I am able to wait to act on my emotions.	-.04	-.44	**-.51**
44. People tell me I am good at expressing my emotions.	-.25	-.46	**.50**
73. I am more comfortable 'talking around' emotions I am feeling, rather than talking about them directly.	-.03	.21	**-.48**
54. Sometimes it is good to keep my emotions to myself.	-.10	.06	**-.47**
4. I am quick to act on my emotions.	-.19	.14	**.45**
**Note:** *N* = 2,840. The largest loading for each item is highlighted in bold.			
